# Estimation of the impact of hospital-onset SARS-CoV-2 infections on length of stay in English hospitals using causal inference

**DOI:** 10.1186/s12879-022-07870-w

**Published:** 2022-12-09

**Authors:** James Stimson, Koen B. Pouwels, Russell Hope, Ben S. Cooper, Anne M. Presanis, Julie V. Robotham

**Affiliations:** 1 HCAI, Fungal, AMR, AMU and Sepsis Division, UK Health Security Agency, London, UK; 2 NIHR Health Protection Research Unit in Healthcare Associated Infections and Antimicrobial Resistance at University of Oxford in Partnership with the UK Health Security Agency, Oxford, UK; 3 Health Economics Research Centre, Nuffield Department of Population Health, University of Oxford, Oxford, UK; 4 Oxford Centre for Global Health Research, Nuffield Department of Medicine, University of Oxford, Oxford, UK; 5 MRC Biostatistics Unit, School of Clinical Medicine, University of Cambridge, Cambridge, UK; 6 Joint Modelling Team, UK Health Security Agency, London, UK

**Keywords:** COVID-19, Public health data, Excess length of stay, Causal inference

## Abstract

**Background:**

From March 2020 through August 2021, 97,762 hospital-onset SARS-CoV-2 infections were detected in English hospitals. Resulting excess length of stay (LoS) created a potentially substantial health and economic burden for patients and the NHS, but we are currently unaware of any published studies estimating this excess.

**Methods:**

We implemented appropriate causal inference methods to determine the extent to which observed additional hospital stay is attributable to the infection rather than the characteristics of the patients. Hospital admissions records were linked to SARS-CoV-2 test data to establish the study population (7.5 million) of all non-COVID-19 admissions to English hospitals from 1st March 2020 to 31st August 2021 with a stay of at least two days. The excess LoS due to hospital-onset SARS-CoV-2 infection was estimated as the difference between the mean LoS observed and in the counterfactual where infections do not occur. We used inverse probability weighted Kaplan–Meier curves to estimate the mean survival time if all hospital-onset SARS-CoV-2 infections were to be prevented, the weights being based on the daily probability of acquiring an infection. The analysis was carried out for four time periods, reflecting phases of the pandemic differing with respect to overall case numbers, testing policies, vaccine rollout and prevalence of variants.

**Results:**

The observed mean LoS of hospital-onset cases was higher than for non-COVID-19 hospital patients by 16, 20, 13 and 19 days over the four phases, respectively. However, when the causal inference approach was used to appropriately adjust for time to infection and confounding, the estimated mean excess LoS caused by hospital-onset SARS-CoV-2 was: 2.0 [95% confidence interval 1.8–2.2] days (Mar-Jun 2020), 1.4 [1.2–1.6] days (Sep–Dec 2020); 0.9 [0.7–1.1] days (Jan–Apr 2021); 1.5 [1.1–1.9] days (May–Aug 2021).

**Conclusions:**

Hospital-onset SARS-CoV-2 is associated with a small but notable excess LoS, equivalent to 130,000 bed days. The comparatively high LoS observed for hospital-onset COVID-19 patients is mostly explained by the timing of their infections relative to admission. Failing to account for confounding and time to infection leads to overestimates of additional length of stay and therefore overestimates costs of infections, leading to inaccurate evaluations of control strategies.

## Background

The first confirmed cases of novel coronavirus Severe Acute Respiratory Syndrome Coronavirus 2 (SARS-CoV-2) in late January 2020 were soon followed in early March 2020 with the earliest cases of suspected hospital-onset infections, as reflected in our source data. In the first wave of the pandemic in England, it has been reported that up to 1 in 6 SARS-CoV-2 infections in hospitalised patients could be attributed to in-hospital acquisition [1] based on confirmed laboratory tests at least 8 days into a hospital spell. Consistent with this report, it is estimated [2, 3] that approximately 20–25% were likely nosocomial when additionally accounting for likely missed (that is, never detected by a PCR test and recorded) infections. Up to the end of August 2021, 97,762 possible hospital-onset infections were recorded in English hospitals, comprising roughly 0.5% of admitted patients. It is important to understand the impact that these infections have had on the length of stay in hospital (LoS) because of both the financial cost to the National Health Service, potential negative impact on the patients themselves, and the negative repercussions for the ability to treat other conditions. Excess length of stay is important for estimating the cost of infection, a key parameter in cost-effectiveness evaluations of interventions. This informs the decisions of policy makers, in particular relating to the burden associated with increased occupancy of hospital beds and associated costs.

We observe that LoS is substantially higher for patients who tested positive for SARS-CoV-2 during a spell in hospital, as compared to non-COVID-19 admissions. The estimation of hospital LoS associated with COVID-19 infections has been discussed in a UK setting [4]; and LoS distributions have been estimated in an international context [5]. However we are not aware of any studies specifically examining the LoS of nosocomially infected patients, or attempting to explain the excess LoS caused by, or attributed to, SARS-CoV-2 infections. There are studies which attribute excess LoS for other healthcare associated infections using a variety of methods—for example, assessing the global burden of antimicrobial resistant infections [6, 7] and assessing the economic burden of bloodstream infection in Europe [8] using multistate models.

The approach we take, using inverse probability weighted survival curves [9] to estimate excess LoS, avoids some of the pitfalls which can be encountered in analyses where time-dependency is a factor [10]. Time from admission to infection is important for two reasons: time already spent in hospital means that the appropriate population for comparison has also to have spent that amount of time in hospital, and furthermore the make-up of the comparison population with respect to confounding variables shifts over time. Any attempt to assess the impact of a nosocomial infection must take into account the timing of that infection relative to the patient’s admission date as this time represents the extent to which the patient is exposed to the risk of acquiring the infection and is crucial in determining the population of hospital patients to whom the infected patient should be compared—for example, patients who acquire their infection 10 days after admission are likely to have very different characteristics to those who acquire their infection after 2 days, given that a large proportion of patients will have been discharged during that time, avoiding further exposure.

The aim of this study is to evaluate the average impact of acquiring a SARS-CoV-2 infection during a hospital spell on a patient’s length of stay. We make use of a methodology for estimating excess LoS which takes account of the timing of infection relative to admission and adjusts for baseline and time-varying confounding [9].

## Methods

### Data

Data on all SARS-CoV-2 PCR tests from laboratories across England undertaking Pillar 1 and Pillar 2 testing from January 2020 until the end of August 2021 were obtained from the United Kingdom Health Security Agency’s Second Generation Surveillance System (SGSS) [11]. Pillar 1 tests were carried out in Public Health England (PHE) labs and National Health Service (NHS) hospitals for patients and health and care workers, whereas Pillar 2 tests were conducted for the wider population, e.g. at walk-in testing sites. For people with multiple SARS-CoV-2 positive tests, the earliest positive test date was retained.

Data on all hospital admissions in England were obtained from the Secondary Uses Service (SUS) [12]. From SUS, we constructed patient hospital spells made up of contiguous episodes at a single hospital trust (SUS data are presented in consultant episodes, where a patient is under the continuous care of a single consultant). These data contain information on admission and discharge dates, whether routine or emergency admission, age, sex, ICD-10 codes (used for defining a measure of comorbidities using the comorbidity R package, version 0.5.3), and surgical interventions. Data used in this analysis were extracted on Feb 20th 2022 and contained admissions from the beginning of March 2020 through to the end of August 2021. Records with missing patient spell identifiers were excluded, as were records with no discharge date recorded or where the discharge date was apparently before the admission date. In total some 2% of raw records were thus filtered out. Spells of less than 2 days in length were excluded as they are not relevant under our definition of potential nosocomial cases. Patients who had tested positive prior to admission, or within the first two days following hospitalisation, were removed from the risk set.

**Fig. 1:**
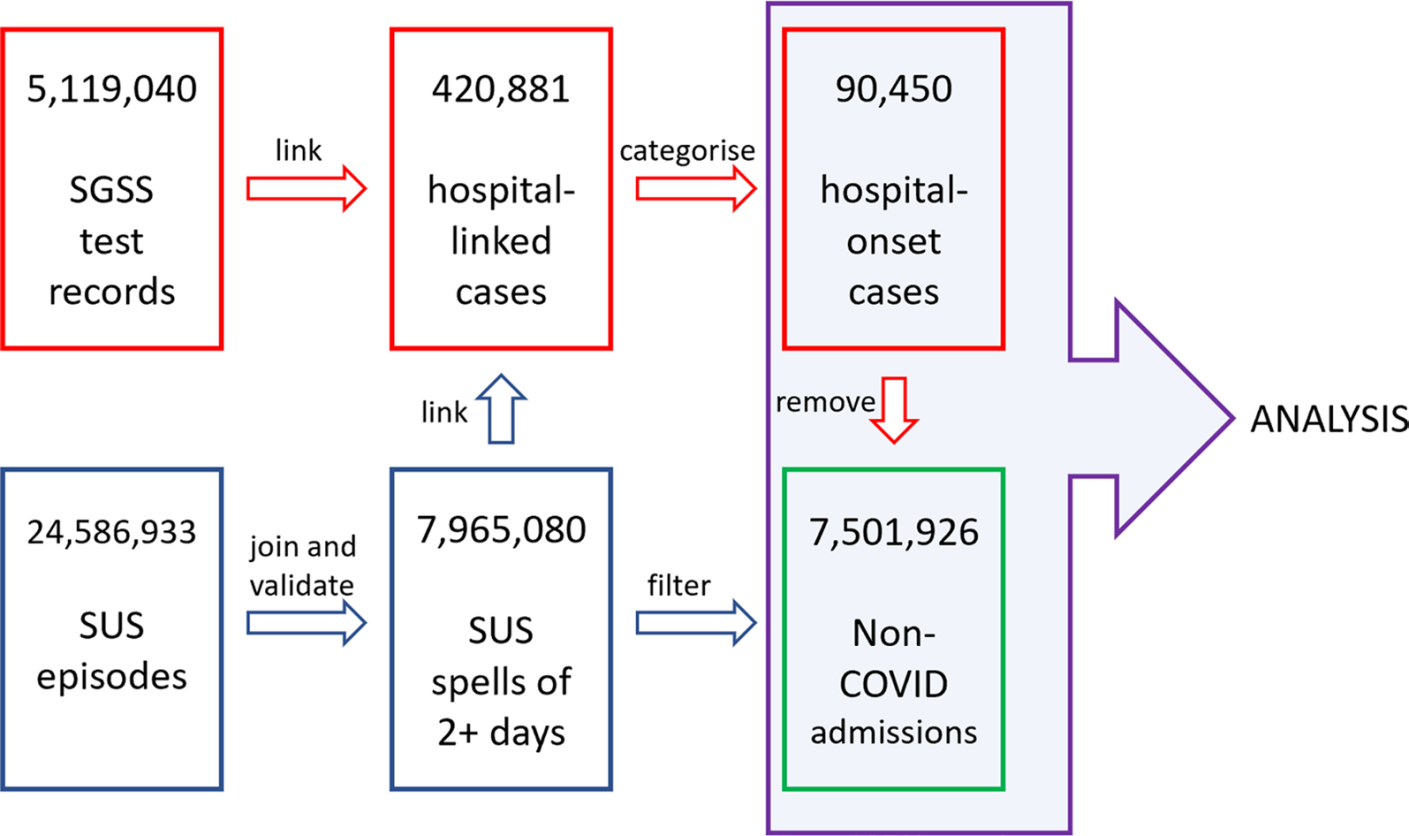
Data flow summary

SARS-CoV-2 PCR test data were linked to SUS admissions data via patient NHS number where available or using an exact match on both date of birth and local patient identifier where NHS number was not available. Hospital-linked cases are defined as those where the first positive test date occurs whilst a patient is in hospital, within 14 days prior to admission, or with 14 days following discharge. A summary of the data flows is shown in Fig. 1.

Records with implausible ages (over 115 years) were removed. Missing hospital spell identifiers mean that up to 0.5% of hospital spells may not have been accurately built up from their component episodes. Missing spell end dates resulted in 0.1% of spells being excluded altogether.

All time-related information relating to admissions, discharges and PCR tests was available only to the nearest day. When a patient dies in hospital, this is counted as a discharge with discharge date equal to date of death. Wherever we refer to infection times, it should be understood to mean the number of days from admission date to the first detection of SARS-CoV-2 infection. First positive test (specimen date) therefore serves as a proxy for infection date.

We included sex, age, Charlson comorbidity index (based on ICD10 codes), month of admission and admission method (elective/non-elective) as baseline confounders. Whether a patient has had invasive surgical procedures is a possible time-varying confounder and is included. Where applicable, that is for phases 3 and 4, whether or not the patient had been double-vaccinated 14 days or more before admission was additionally included at baseline. These risk factors are all potential confounders given that they may influence both the length of stay and the risk of acquiring a SARS-CoV-2 infection.

LoS is calculated based on the admission and discharge dates of the hospital stay within which the positive test occurs. Subsequent re-admissions do not count toward LoS unless the re-admission date falls on the previous discharge date, in which case the stays are joined together. We do not include reinfections or reactivations of disease and assume that these are relatively small in number, though the full picture likely changes over time and is not fully understood at the time of writing [13].

### Scope

**Fig. 2:**
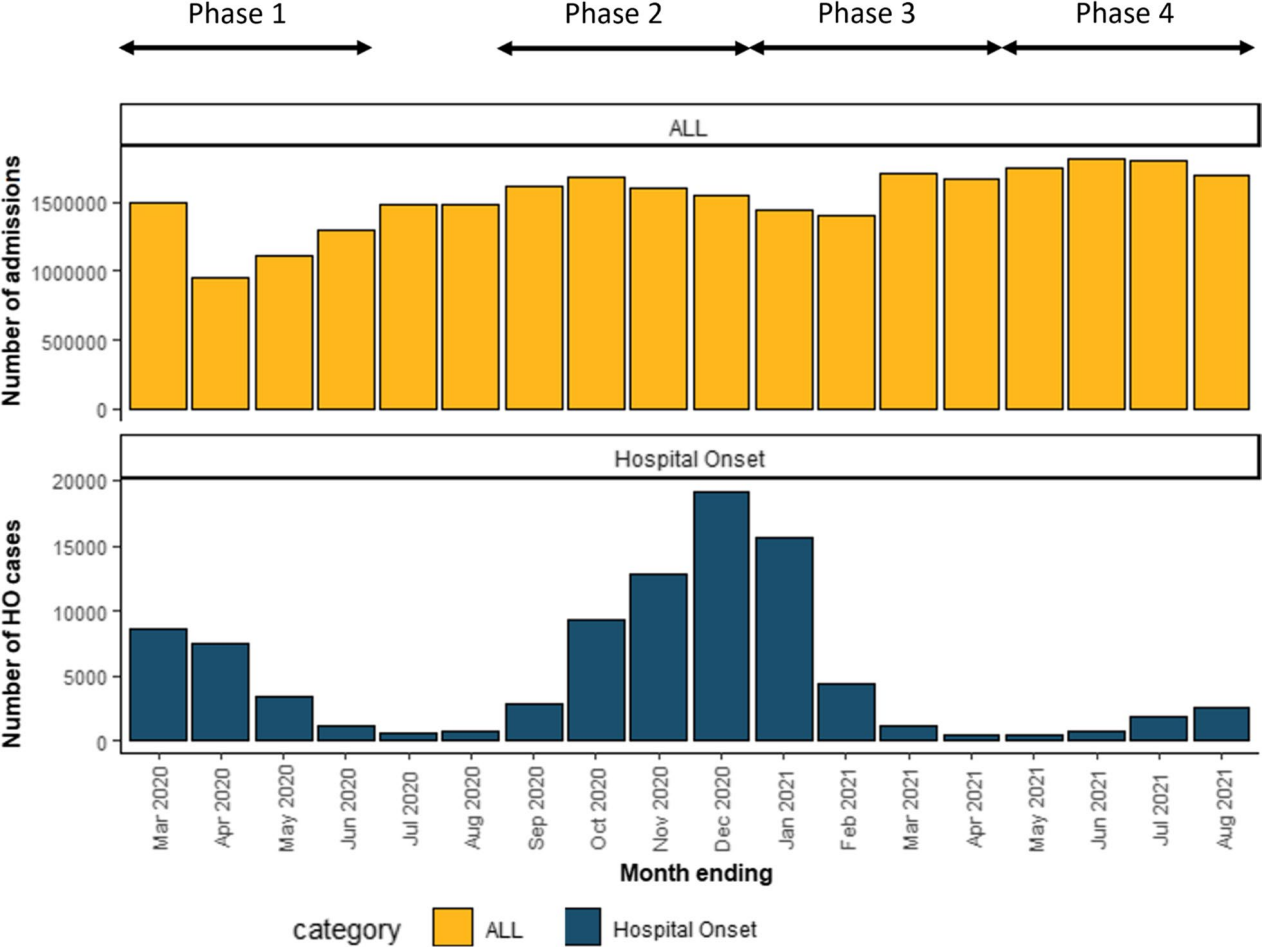
Phases

The analysis is split into four distinct phases (see Fig. 2) in order to examine the impact of COVID-19 at stages of the pandemic differing with respect to overall case numbers, the vaccine rollout and prevalence of variants. Phase 1: March 2020 through June 2020 inclusive, consists of most of the first wave. Phase 2: September 2020 through December 2020 inclusive, consists of the earliest part of the second wave before large numbers of people received a first vaccine dose. Phase 3: January 2021 through April 2021, consists of the remaining part of the second wave where increasing numbers of patients had been vaccinated, and when the Alpha variant was dominant. Phase 4: May 2021 through August 2021, consists of the third wave, when the Delta variant was dominant.

### Analysis

We define a COVID-19 infection as hospital-onset if the patient’s first positive specimen date is at least two days after admission and does not occur after discharge. This definition includes possible, probable and definite hospital-onset cases, as described in [1]. The study population includes all patients admitted to English hospitals between March 1st 2020 and August 31st 2021 who stayed at least 2 days in hospital, excluding community-onset COVID-19 cases. Note that the latter includes those whose first positive specimen date is on the day of admission or on the day after; this is because these infections were very likely acquired before admission.

LoS is calculated in days as discharge date minus admission date, regardless of whether the discharged patient was alive or dead on discharge. Since we are interested in time to discharge—dead or alive—as the outcome, there is no competing risk between discharge and death. We estimate survival probabilities up to day 60 in hospital for the observed study population, comprising both infected (hospital onset infections) and uninfected cohorts, using a standard Kaplan–Meier analysis. We sum these probabilities over the days up to day 60 to obtain the average LoS up to day 60, the restricted mean survival time [14]. Applying this restriction avoids including long-staying patients in the study where the low numbers of patients provides insufficient support to adjust accurately for time-varying confounding. Approximately 3% of cases in our dataset have LoS of more than 60 days.

We then estimate what the LoS would have been in the counterfactual scenario where the infected did not acquire the infection, following the methodology described in [9]. To estimate the counterfactual LoS, cases are censored on the day of infection, so that their observed LoS after infection does not contribute to the LoS estimate. Furthermore, inverse probability weights were used to account for the potential informative censoring introduced by treating SARS-CoV-2 infections as censoring events. The weights remove confounding by baseline and time-varying confounders by rebalancing the case contributions on each of the 60 days; they were constructed using pooled logistic regression models for the probability of infection on each day [15, 16], so for each patient each day in hospital is treated as separate observation.

As discussed above, potentially important risk factors were selected for inclusion as variables in the model. Continuous variables were modelled as cubic splines with degrees of freedom chosen to minimise the Akaike Information Criterion: age was modelled by a cubic spline with 5 degrees of freedom; Charlson comorbidity with 2 degrees of freedom. Interactions between age and comorbidity were considered, but found not to improve the fit.

The excess LoS is estimated as the difference between the mean LoS observed and the counterfactual mean LoS. The excess LoS per infected case is obtained by multiplying this difference in means by the total number of patients and dividing by the number of patients whose infection was detected within the first 60 days of their stay. We estimated 95% confidence intervals assuming that the weights are deterministic ([9], supplement). We tested that this assumption is appropriate by re-sampling the coefficients of the pooled regression model, and re-calculating the weights based on these coefficients, to verify that the uncertainty in the weights was small relative to the uncertainty of the regression model.

### Re-admissions

For the main analysis, re-admissions following the spell in which the SARS-CoV-2 infection was detected were not considered. To explore the possible impact of including re-admission in the length of stay calculations, we conducted an analysis for Phase 1. To achieve this, we additionally included any further hospital admissions for which the admission date was within 7 days following the discharge date of the initial hospital-onset stay. Additional days spent in hospital were added to the total LoS and treated as if a single continuous spell in hospital; days between discharge and re-admission are thus ignored.

### Sensitivity to hospital-onset definition

We carried out an analysis of the impact of altering the definition of hospital-onset from those cases that are detected at least 2 days following admission to 7 days and 14 days following admission. These correspond to alternative definitions based on the likelihood of the infection being acquired in hospital as used, for example, in [1]. The 3 definitions can be loosely interpreted as covering all (detected) possible hospital-acquired infections (2 days or more), those that are probably or almost certainly hospital-acquired (7 days or more), and those that are almost certainly hospital-acquired (14 days or more).

### Simulations

We tested simulated scenarios to validate the implementation of the methodology. Firstly, an analysis was run on a sample of the data to obtain an estimated excess LoS. An extra day was added to the discharge date of all hospital-onset cases in this sample and the analysis was re-run, resulting in an increase of one day to the estimated excess LoS. Secondly, a sample set was created where the hospital-onset cases had the same characteristics and LoS as the non-COVID-19 cases, and it was confirmed that the model returned no excess, as expected. In both examples, further additional days were added to hospital-onset discharge dates, with the expected result on the additional excess obtained.

### Software

All analyses were carried out using R version 4.0.3.

## Results

In Table 1 we summarise the characteristics of interest of admissions leading to hospital stays of 2 or more days. Vaccination status only becomes relevant in phases 3 and 4. We found that the number of admissions varied significantly over the phases (see also Fig. 2), falling early in Phase 1 compared to pre-pandemic levels [17], and rising over time thereafter with a smaller fall again following the peak of the second wave in late 2020.

**Table 1:** Summary of admissions resulting in hospital stays of at least 2 days

	Phase 1 Mar–Jun 2020		Phase 2 Sep–Dec 2020		Phase 3 Jan–Apr 2021		Phase 4 May–Aug 2021	
	All	HO COVID	All	HO COVID	All	HO COVID	All	HO COVID
Admissions, number	1,001,614	19,530	1,159,770	41,771	1,145,645	20,055	1,349,902	5,119
Sex female, number (%)	555,539 (55)	9,093 (47)	648,956 (56)	20,656 (49)	639,273 (56)	9,982 (50)	752,179 (56)	2,386 (47)
Age, median years (IQR)	63 (33–79)	79 (68–86)	63 (34–79)	79 (69–87)	64 (35–79)	79 (68–86)	64 (35–79)	75 (58–84)
Charlson score, median (IQR)	1 (0–2)	2 (1–3)	1 (0–2)	2 (1–3)	1 (0–2)	2 (1–3)	1 (0–2)	1 (0–3)
Emergency admission, number (%)	821,322 (82)	17,950 (92)	911,894 (79)	38,755 (93)	923,915 (81)	18,916 (94)	1,063,179 (79)	4,643 (91)
Had surgery, number (%)	98,304 (10)	1,351 (7)	151,515 (13)	3,459 (8)	136,280 (12)	1,594 (8)	177,604 (13)	348 (7)
Double-vaccinated 14 days before admission, number (%)					74,030 (6)	222 (1)	800,616 (59)	3,571 (70)

**Fig. 3:**
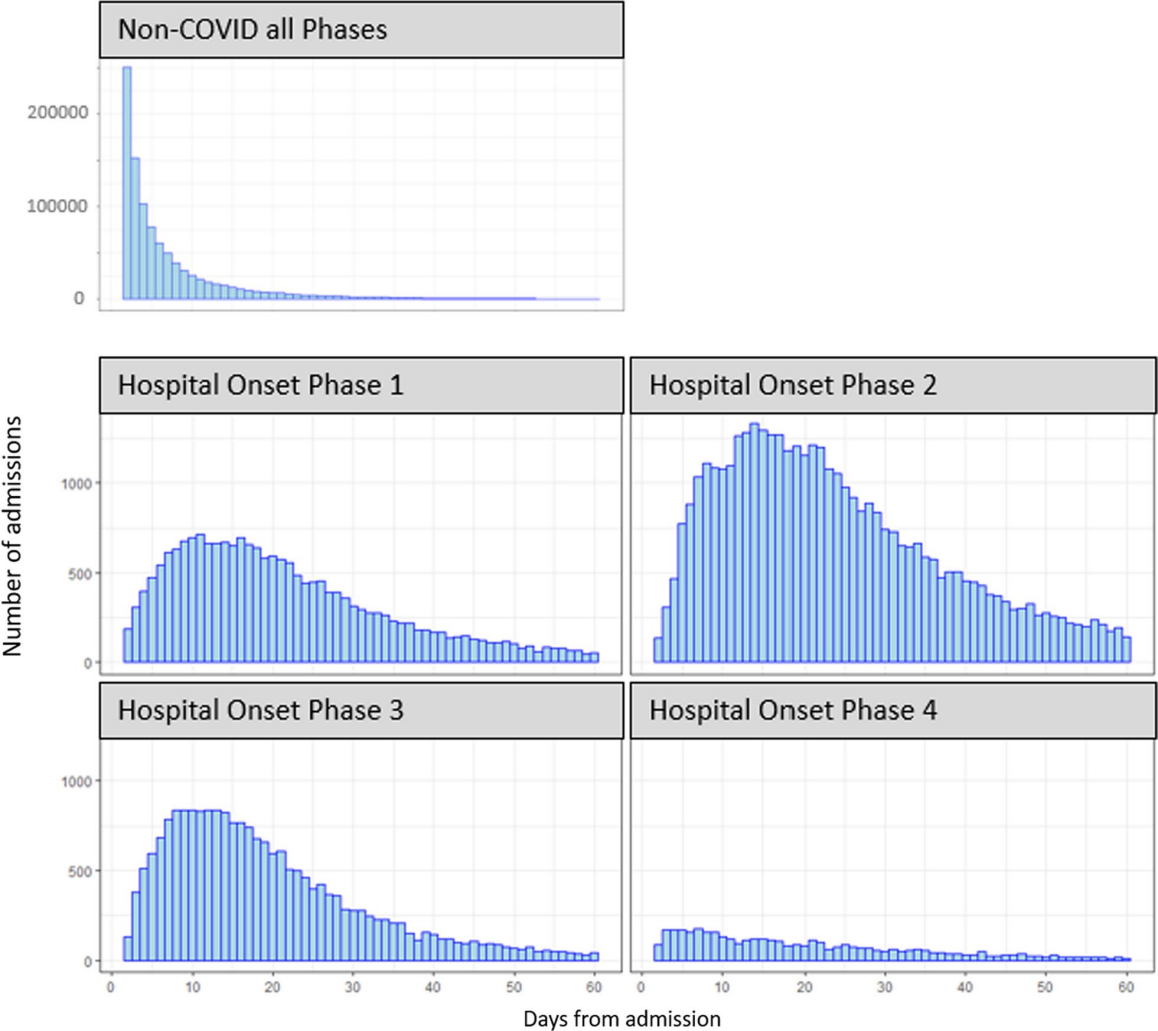
LoS distribution for all 4 phases, truncated at 60 days

**Fig. 4:**
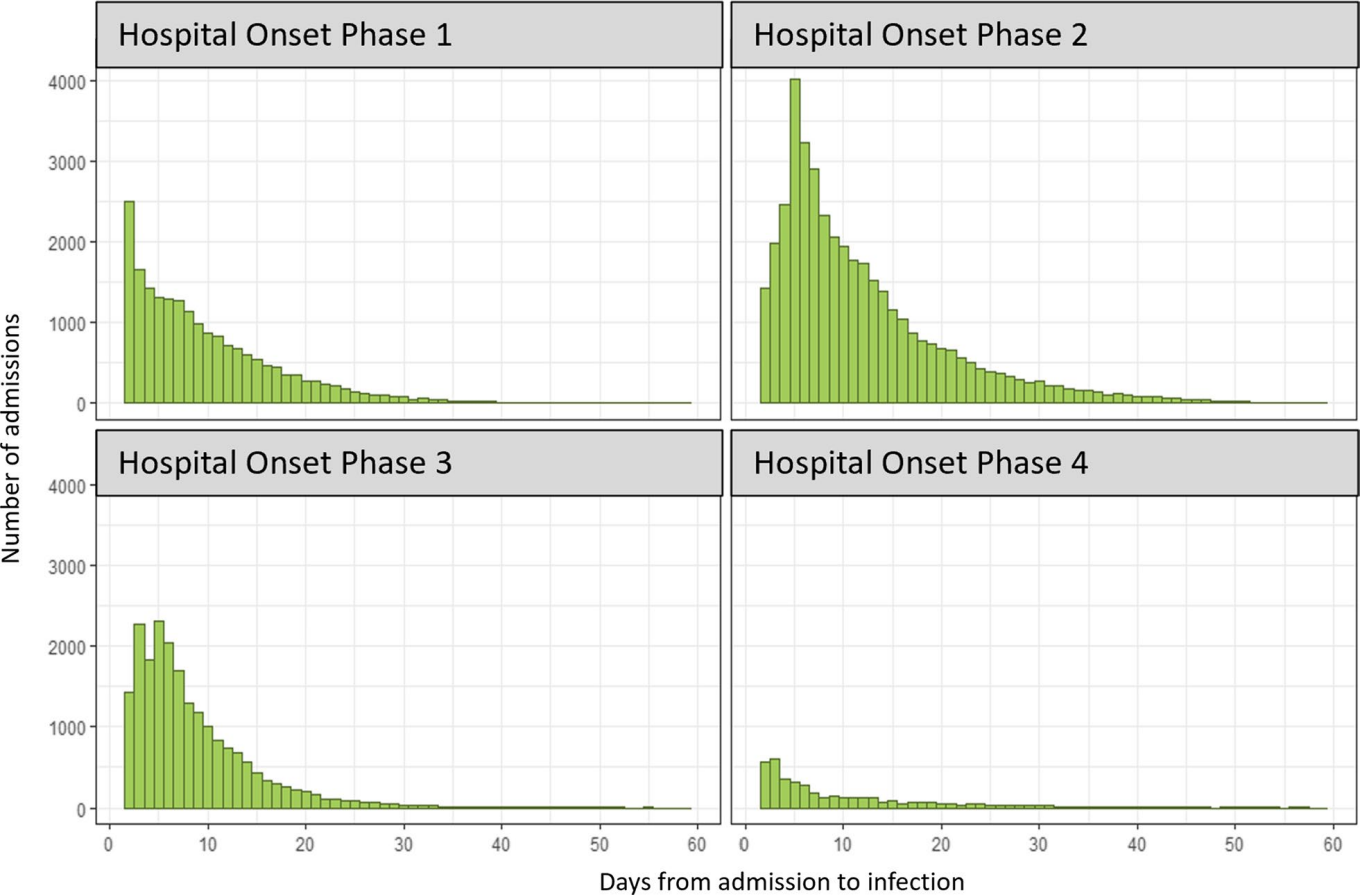
LoS distribution of time from admission to first positive test in each phase, truncated at 60 days

**Fig. 5:**
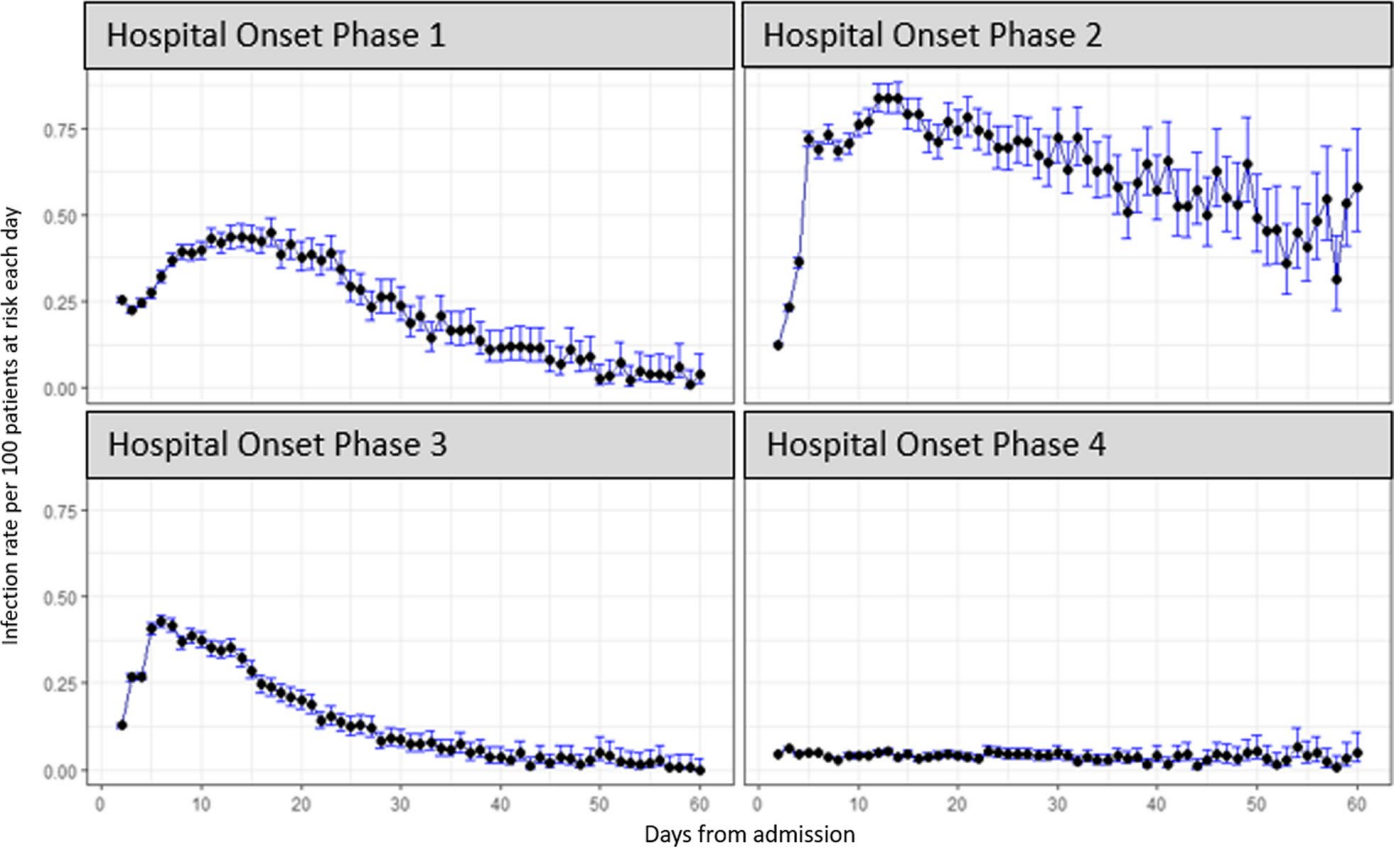
Detected infection rates for each phase from day 2 to day 60 of hospital stay, with 95% confidence intervals based on Wilson score intervals

The distribution of observed LoS is highly skewed, with most patients having a relatively short LoS (Fig. 3). This is for all the admissions considered in this study, so stays of 0 and 1 day are not included, and the profile is very similar in each of the phases (not shown). However, for hospital-onset COVID-19 cases the distribution varies across the phases. The pattern of infection is more markedly different across the phases, as reflected in Figs. 4 and 5; infection rates were relatively high in phase 2 and relatively low in phase 4. Rates were confirmed to be significantly different pairwise between all phases (p < 0.001) using post-hoc Chi-squared with Bonferroni correction.

In all phases the observed average LoS was considerably higher for hospital-onset COVID patients than for uninfected patients, as set out in Table 2.

**Table 2:** Observed length of stay observed over the 4 phases, for patient stays of at least 2 days

	Phase 1 Mar–Jun 2020			Phase 2 Sep–Dec 2020			Phase 3 Jan–Apr 2021			Phase 4 May–Aug 2021		
	All	HO COVID	Non-COVID	All	HO COVID	Non-COVID	All	HO COVID	Non-COVID	All	HO COVID	Non-COVID
Median LoS, days (IQR)	4 (3–9)	20 (12–32)	4 (2–9)	4 (2–9)	24 (15–39)	4 (2–8)	5 (3–10)	18 (11–28)	5 (3–9)	5 (3–9)	24 (12–46)	5 (3–9)
Mean LoS, days	8.102	24.14	7.783	8.358	28.08	7.621	8.502	21.77	8.265	8.454	29.04	8.375

Using the methodology described in the Methods section, estimates were obtained (Table 3) of the expected length of stay in the counterfactual scenario where infection did not occur.

**Table 3:** Summary of results showing the estimated excess LoS due to hospital-onset SARS-COV-2 infection, with and without confounding adjustment using inverse probability weights

	Phase 1 Mar–Jun 2020	Phase 2 Sep–Dec 2020	Phase 3 Jan–Apr 2021	Phase 4 May–Aug 2021
Observed mean LoS, days	8.102	8.358	8.502	8.454
Unadjusted counterfactual^a^ mean LoS, days	8.076	8.322	8.488	8.448
Implied excess days per infection (95% CI)	1.3 (1.1, 1.5)	1.0 (0.8, 1.1)	0.8 (0.6, 1.0)	1.5 (1.1, 1.9)
Counterfactual^a^ mean LoS, adjusted for confounding, days	8.063	8.309	8.486	8.448
Implied excess days per infection (95% CI)	2.0 (1.8, 2.2)	1.4 (1.2, 1.6)	0.9 (0.7, 1.1)	1.5 (1.1, 1.9)

^a^Counterfactual where infection does not occur

Because inverse probability weighting removes confounding by creating a pseudo-population in which the probability of infection is independent of the measured confounders, it is desirable for the stability of the results that the weights are not too unbalanced (that is, far away from 1), which can occur when there are individuals in the population with very low probability of infection. The inverse probability weights had a mean of 0.999 and median 1.000, with interquartile range of 0.011. The entire range was 0.15–133, weights above 10 being capped at 10 to ensure stability.

The implied excess LoS decreases from 2.0 days down to 0.9 days over the first 3 phases, but increases again to 1.5 in phase 4. Even when not adjusted for confounding, the excess LoS due to infection (Table 3) is substantially lower than the apparent difference observed (Table 1) for all four phases. For the counterfactual where no infections occur, there is a notable difference in implied excess depending on whether we account for confounding or not in phase 1, but over later phases the effect of adjusting for confounding diminishes.

If we consider alternative definitions of hospital-onset (Table 4) in phase 1, we see that as we make the definition stricter, the implied excess LoS falls from 2.0 to 1.5 days. Meanwhile, the effect of adjusting for confounding is seen to increase the stricter the definition.

**Table 4:** Sensitivity to hospital-onset definition during phase 1

Cut-off	2 days	7 days	14 days
Observed mean LoS, days	8.102	16.903	27.423
Unadjusted counterfactual^a^, mean LoS, days	8.076	16.894	27.460
Implied excess days per infection	1.3	0.2	− 1.0
Counterfactual^a^ mean LoS, adjusted for confounding, days	8.063	16.842	27.366
Implied excess days per infection	2.0	1.8	1.5

^a^Counterfactual where infection does not occur

Re-admissions within 7 days occurred for approximately 15% of hospital onset COVID-19 patients and 12% of non-COVID-19 patients. Including re-admissions increases the observed mean by about 1 day (Table 5) but has little effect on the excess days per infection (though increasing the excess by 0.6 days when not adjusting for confounding).

**Table 5:** Summary of results showing the estimated excess LoS including re-admissions due to hospital-onset SARS-COV-2 infection, with and without confounding adjustment using inverse probability weights

Phase	Phase 1 Mar–Jun 2020
Observed mean LoS, days	9.048
Unadjusted counterfactual^a^ mean LoS, days	9.010
Implied excess days per infection (95% CI)	1.9 (1.7, 2.1)
Counterfactual^a^ mean LoS, adjusted for confounding, days	9.007
Implied excess days per infection (95% CI)	2.0 (1.8, 2.2)

^a^ Counterfactual where infection does not occur

## Discussion

We would expect an infection with SARS-COV-2 to prolong a hospital patient’s stay for various reasons. Once infected the patient may have stayed long enough to develop COVID-19 of sufficient severity to warrant being kept longer in hospital. Even in cases not reaching a high level of severity, if infection was thought to be of sufficient additional concern patient discharge could have been delayed. Delays are also expected to have occurred in discharging known SARS-COV-2 patients to a care home or other form of community care [18]. Conversely, infection might shorten stay due to hospitals making efforts to discharge SARS-COV-2 patients early to reduce risks of transmission, or because infected patients died prematurely as a result of the infection. For this last reason, irrespective of the size of the excess LoS, the consequences of acquiring SARS-CoV-2 in hospital were severe. This study does not tell us anything about each of these individual processes or how they may have contributed to the results.

The variation in excess LoS over the four phases (Table 3) has many potential explanations. How much these changes were due to factors such as testing regimes in hospitals [19, 20], the roll-out of the vaccine programme, immunity caused by previous infection, prevalence of the SARS-COV-2 in the community, and improved infection control measures in the healthcare system [21, 22], is difficult to assess. The excess is highest in the first phase, as might be expected as there was little experience in treating COVID-19, leading to increased length of stay associated with less favourable patient outcomes. The excess LoS fell over the first 3 phases, consistent with the increasing availability of effective treatments [23], but then rose again in the fourth phase, suggesting other factors were at play. Hospital-onset infection rates (Fig. 5) were highest in phase 2, but very low in Phase 4, not correlating with the excess LoS.

The time from infection to having symptoms possibly requiring hospitalisation [24] means that a proportion of hospital-onset COVID-19 patients were discharged and subsequently re-admitted. However, since there is a slightly lower but comparable re-admission rate in the general patient population, this effect is not as large as might be expected (see Tables 1, 5).

Considering alternative definitions of hospital-onset (Table 4) shows that the implied excess LoS is lower when considering patients who have already spent a greater amount of time in hospital before infection. One possible explanation is that the patients who already have a substantial length of stay already have a condition which requires lengthy treatment in hospital, so the acquiring of a SARS-COV-2 infection has a lesser marginal effect. The fact that the effect of adjusting for confounding is higher in longer staying patients also points to the association of certain groups (e.g., older patients, who are over-represented amongst longer stayers) with increasing comorbidities and a consequent greater impact of any infection.

A proportion of the hospital-onset infections will have been acquired in the community before admission to hospital, especially for infections which occur in the first few days. When drawing any conclusions on the effect of these infections it should be understood that it is not assumed that transmission necessarily occurred in the hospital setting.

There are some limitations to this study. Advice to routinely test all non-elective admissions at admission time was not given until late April 2020 [19], and advice to test again after 5–7 days was given even later [20], well into the first phase, so the use of first positive test date as a proxy for infection date would have been less accurate early in the pandemic. Thus the possibility of mis-attributing cases as hospital-onset may have been more likely during the first wave before routine testing was fully introduced as patients are more likely to have been admitted already with infection, but only tested once severe COVID-typical symptoms started to appear. Late detection of infections may also have led to mis-classification and under-estimation of excess LoS: if infections are detected late, some days in hospital will be counted as uninfected while in reality being infected. A proportion of SARS-COV-2 infections will have remained undetected, either because of the aforementioned lack of routine testing in the early stages of the pandemic or because of false negative tests; this study measures the effect of detected cases only.

Hospital spells do not appear in the SUS data until after they have completed; even though we only measure length of stay up until 60 days after admission, there may be long-running spells which are missing from our data. For all except phase 4, these spells would have to be several months long due to the time elapsed between the period being studied and the extraction date used for the analysis and are likely to be of little impact. Only LoS from the current (that in which the positive test fell) hospital stay was considered, resulting in a possible under-estimation of excess LoS. As with any observational study, there is likely to be unmeasured confounding; this is mitigated by the fact that the results indicate that the predominant influence on LoS is the time of infection. Though of considerable interest, we did not look specifically at mortality as part of this study. We do not have re-infection tests in our data, so that if someone is re-infected while in hospital, we would inadvertently classify them as uninfected instead of infected. This might bias the results if their LoS is lengthened by being re-infected, but for the time period studied we expect the impact to have been slight.

## Conclusions

Hospital-onset SARS-CoV-2 caused a small but notable excess LoS in English hospitals. The much higher LoS observed for hospital-onset COVID-19 patients is for the most part explained by the timing of their infections – they were in general already relatively long-stayers in hospital *before* they acquired their infections, the mean time between admission and first positive test being 8 days. Although the excess LoS is relatively small, this does not mean that the consequences of acquiring SARS-CoV-2 in hospital were not severe. In total, the excess number of days equates approximately to an extra 130,000 bed days. Assuming a typical hospital trust capacity of 500 beds [25], that is equivalent to over half a years’ worth of a trust’s bed capacity.

The methodology we have used in this study reinforces the importance of choosing appropriate methods in situations where the effect of infections may depend on their timing. Our estimated additional LoS caused by hospital-onset COVID-19 infections of less than 2 days is in stark contrast to the observed difference in restricted mean LoS of 15 days (comparing those with and without nosocomially acquired COVID-19). As illustrated above, in this study by far the most important factor explaining the difference in observed length of stay (timing of infection) can be discerned simply by carrying out appropriate survival analyses. The more sophisticated inverse probability weighting techniques (to adjust for confounders) further refined the results obtained, but were of secondary impact on the results. Nevertheless they (or equivalent methods) are vital in any analysis which seeks to correctly account for time-varying confounding; failing to do this leads to overestimates of additional length of stay and therefore overestimates costs of infections, leading to inaccurate evaluations of control strategies.

## Data Availability

All data were collected within statutory approvals granted to United Kingdom Health Security Agency for infectious disease surveillance and control. Information was held securely and in accordance with the Data Protection Act 2018 and Caldicott guidelines. The data that support the findings of this study are available from NHS Digital but restrictions apply to the availability of these data, which were used under license for the current study, and so are not publicly available. Data are however available from the authors upon reasonable request and with permission of NHS Digital.

## Abbreviations

SARS-CoV-2: Severe acute respiratory syndrome coronavirus 2
LoS: Length of stay
SGSS: Second generation surveillance system
PHE: Public Health England
NHS: National Health Service
SUS: Secondary Uses Service
